# NaviGO: interactive tool for visualization and functional similarity and coherence analysis with gene ontology

**DOI:** 10.1186/s12859-017-1600-5

**Published:** 2017-03-20

**Authors:** Qing Wei, Ishita K. Khan, Ziyun Ding, Satwica Yerneni, Daisuke Kihara

**Affiliations:** 1 Department of Computer Science, Purdue University, West Lafayette, IN 47907 USA; 2 Department of Biological Science, Purdue University, West Lafayette, IN 47907 USA; 3 Division of Biomedical Statistics and Informatics, Mayo Clinic, Rochester, MN 55905 USA

**Keywords:** Gene function, Gene ontology, GO, Ontology, GO directed acyclic graph, Function similarity, Gene function prediction, GO annotation, function enrichment analysis, GO parental terms, GO association score

## Abstract

**Background:**

The number of genomics and proteomics experiments is growing rapidly, producing an ever-increasing amount of data that are awaiting functional interpretation. A number of function prediction algorithms were developed and improved to enable fast and automatic function annotation. With the well-defined structure and manual curation, Gene Ontology (GO) is the most frequently used vocabulary for representing gene functions. To understand relationship and similarity between GO annotations of genes, it is important to have a convenient pipeline that quantifies and visualizes the GO function analyses in a systematic fashion.

**Results:**

NaviGO is a web-based tool for interactive visualization, retrieval, and computation of functional similarity and associations of GO terms and genes. Similarity of GO terms and gene functions is quantified with six different scores including protein-protein interaction and context based association scores we have developed in our previous works. Interactive navigation of the GO function space provides intuitive and effective real-time visualization of functional groupings of GO terms and genes as well as statistical analysis of enriched functions.

**Conclusions:**

We developed NaviGO, which visualizes and analyses functional similarity and associations of GO terms and genes. The NaviGO webserver is freely available at: http://kiharalab.org/web/navigo.

## Background

Functional elucidation of genes is one of the central problems in modern biology including bioinformatics. For systematic function annotation, GO is widely used as the vocabulary of gene functions [1]. GO terms are arranged in a hierarchical directed acyclic graph (DAG), where parental relationships between terms are represented. GO is updated periodically by the Gene Ontology Consortium [2], and currently holds over 44,000 terms. The DAG structure is divided into three different GO categories (three disconnected roots), namely, Biological Process (BP), Molecular Function (MF), and Cellular Component (CC). The large volume of the vocabulary and their parental relationship make it non-trivial to provide an intuitive summary of GO annotations of genes.

AmiGO [3] is an online tool maintained by the Gene Ontology Consortium [2] that is widely used to search and browse the gene ontology database. Apart from this, there are other existing tools [4–6] that can be used for GO visualization and comparison. QuickGO, a tool that is developed under the Gene Ontology Annotation (GOA) project, allows searches of GO terms and genes with a specified GO, and provides static clickable maps [7]. A drawback of the existing works provide a static way of visualizing GO DAG topology either in a static image or in the SVG format. Once the topologies are generated in the frontend of these servers, users are unable to explore different branches of the GO hierarchy interactively in real-time. Simple tasks of GO terms, for example, listing all the parental terms from query GO terms, or mapping GO terms and visualize them on the GO DAG, are not trivial in the existing web-based tools. Browsing parental GO terms, visualizing GO terms in the DAG topology interactively to find related terms is fundamental in grasping function annotations of genes under studies.

In this work, we present a new interactive web-based tool, NaviGO, for comprehensive analysis of GO terms and gene functions that provide advantages over the existing GO-based web tools in three aspects: first, NaviGO is equipped with an interactive and fast rendering of the GO DAG named GO Visualizer [8], which instantly maps user-input GO terms on the GO DAG. The mapping will color parental terms of user-input GO terms and provide intuitive understanding of the similarity among them (since similarity scores are based on the topology of GO DAGs). On the GO Visualizer, users can interactively expand the hierarchy or change the view, which is advantageous over static pictures offered by AmiGO and other existing GO web tools. Second, we provide an interactive GO relationship analysis and in-depth quantification of GO similarity/divergence by incorporating six scoring schemes. The six scores reflect a variety of relationships of GO terms, ranging from GO topological structure, protein-protein interaction (PPI) association, contextual association, and annotation frequency. Particularly, in NaviGO we have implemented three scoring schemes developed previously in our group for assessing functional coherence of GO terms, namely, Co-occurrence Association Score (CAS), Pubmed Association Score (PAS) [9], and Interaction Association Score (IAS) [10], which are based on statistics of GO term pairs that are observed to co-occur in gene annotations, literature abstracts in PubMed, and physically interacting proteins, respectively. These three scoring schemes enable quantification of GO term distance based on these different contextual associations and provide cross-domain GO term comparison, unlike the three other semantic similarity based scoring schemes (Resnik, Lin and Relevance Similarity [11]; see Methods). Third, leveraging the different GO scoring schemes, in NaviGO we provide quantitative analysis of functional similarities for a group of genes and visualization of functionally similar gene clusters using a scoring scheme of users’ choice. NaviGO is also linked from gene function prediction webservers, PFP [12, 13] and ESG [8, 14], so that function prediction can be readily analyzed.

Besides the exisiting GO visualization and comparison tools mentioned above [3–7], there are other GO-based tools that are more focused on particular biological analysis of genes. GSEA is focused on GO enrichment analysis and linked to a gene annotation database [15]. GeneWeaver is a data repository of genes from multiple species, which includes gene annotations, expressions, QTL, GWAS, and other biological data [16]. Tools associated with GeneWeaver can link a gene dataset to stored data, and compare datasets considering homology and gene overlaps [16]. DAVID stores annotations, domain architectures, and pathway information of genes, and users can classify a gene set which considers common GO annotations of genes [17]. VLAD performs GO enrichment analysis of genes and visualizes results on the GO hierarchy [18]. Compared to these tools, NavGO is unique in that it provides multiple different definitions of GO term similarity and associations, and can visualize and tell parental relationships of GO terms, and also linked from state-of-the-art function prediction servers.

Overall, NaviGO provides tools for exploring and understanding GO vocabulary as a basis of GO function annotation and also offers tools for biological analyses of GO annotations of genes. In addition to the web-based tools, the source codes are made available to download for local use of the software. NaviGO is a useful tool for both computational biologists who deal with GO terms and biologists who perform functional analyses of genes.

## Implementation

NaviGO is a web-based software for analyzing functional similarity and associations of GO terms and genes. NaviGO is equipped with four types of analyses users can perform, which are accessible from each tab of the NaviGO page. They are, “GO Parents” for retrieving and visualizing parental terms of query GO terms, “GO Set” for computing similarity and associations of query GO terms, “GO Enrichment” for identifying enriched GO annotations in a set of query proteins, and “Protein Set” for performing functional similarity and association analysis for a set of query proteins. Each of them is described in details in the following subsections.

**Fig. 1:**
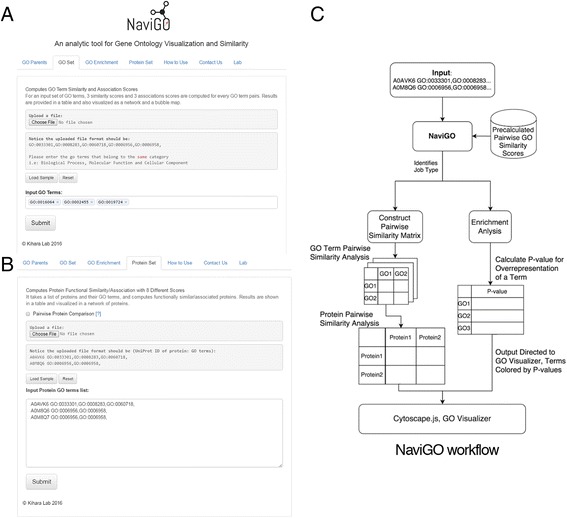
NaviGO input screens and workflow pipeline. **a**, A screenshot of the input page for GO term similarity analysis (GO Set tab). **b**, A screenshot of the protein analysis page (Protein Set tab). **c**, The workflow of NaviGO, which shows how an input dataset of GO terms and gene annotations is processed

The input page and the logical architecture of NaviGO is provided in Fig. 1. NaviGO can either take a list of GO terms for (Fig. 1a) or a list of annotated genes (Fig. 1b) as input of analysis. NaviGO first queries in its underlying MySQL database and retrieves pre-calculated pairwise GO similarity scores computed with the six different scoring schemes. Then, based on the job type, it either constructs similarity matrices based on the input GO terms and further continues to compute functional similarity among gene products/proteins based on the GO similarity matrices or it moves onto performing an enrichment analysis by calculating *p*-values for the overrepresented GO terms in the input. In either case, the final result of these analysis can be visualized by NaviGO’s interactive GO visualizer or in Cytoscape [19].

## Results and discussion

### Real-time and interactive rendering of GO terms

**Fig. 2:**
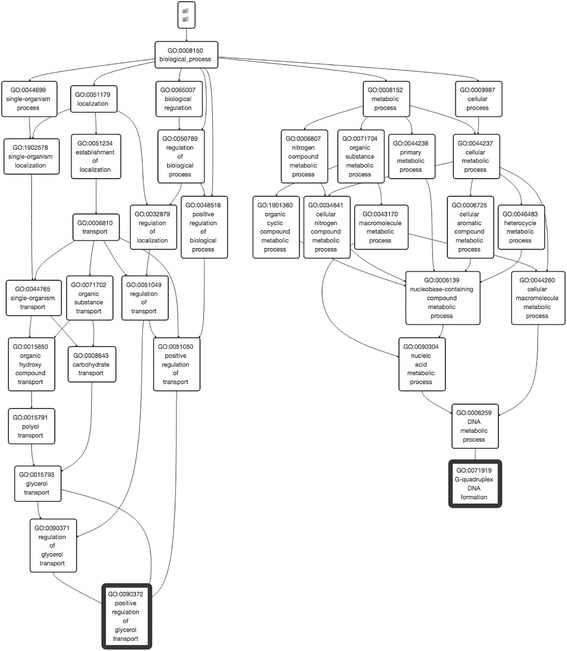
Screenshot of GO visualizer. The GO visualizer GO terms in the GO hierarchy. Common parents of input GO terms that are highlighted with a bold border are shown. GO terms in the visualizer are expandable by clicking a node and layout will be dynamically adjusted when expanded. A static figure of the GO DAG can be downloaded

Retrieving and mapping parental GO terms on the GO hierarchy for query GO terms is implemented as a basic functionality. In the GO Parents page, NaviGO retrieves parental terms for a query and visualizes those GO terms in the DAG interactively (Fig. 2). Users can investigate the relationship of multiple GO terms by looking for their common parent and relative position in the hierarchy. This function is useful for better understanding gene annotations. For example, in UniProt, a number of GO terms are listed as function annotation for a gene, but it is often difficult to understand which terms are closely related and which are not. In such case, visualizing GO annotations on NaviGO can provide clear picture of the annotation.

In the visualization, query GO terms will be circled with bold black in the hierarchy and parental terms for the input GO terms will be listed in the text area so that users can copy and paste them for further use or for writing a document. Branches of the GO DAG can be expanded interactively by clicking a node. Hovering any node in the GO visualization will update the node information on the upper right corner of the frame. To boost the rendering speed, an option is provided that avoids newly expanded nodes from checking and updating parental relationship with the whole set of existing nodes.

### Quantification of GO term association

**Fig. 3:**
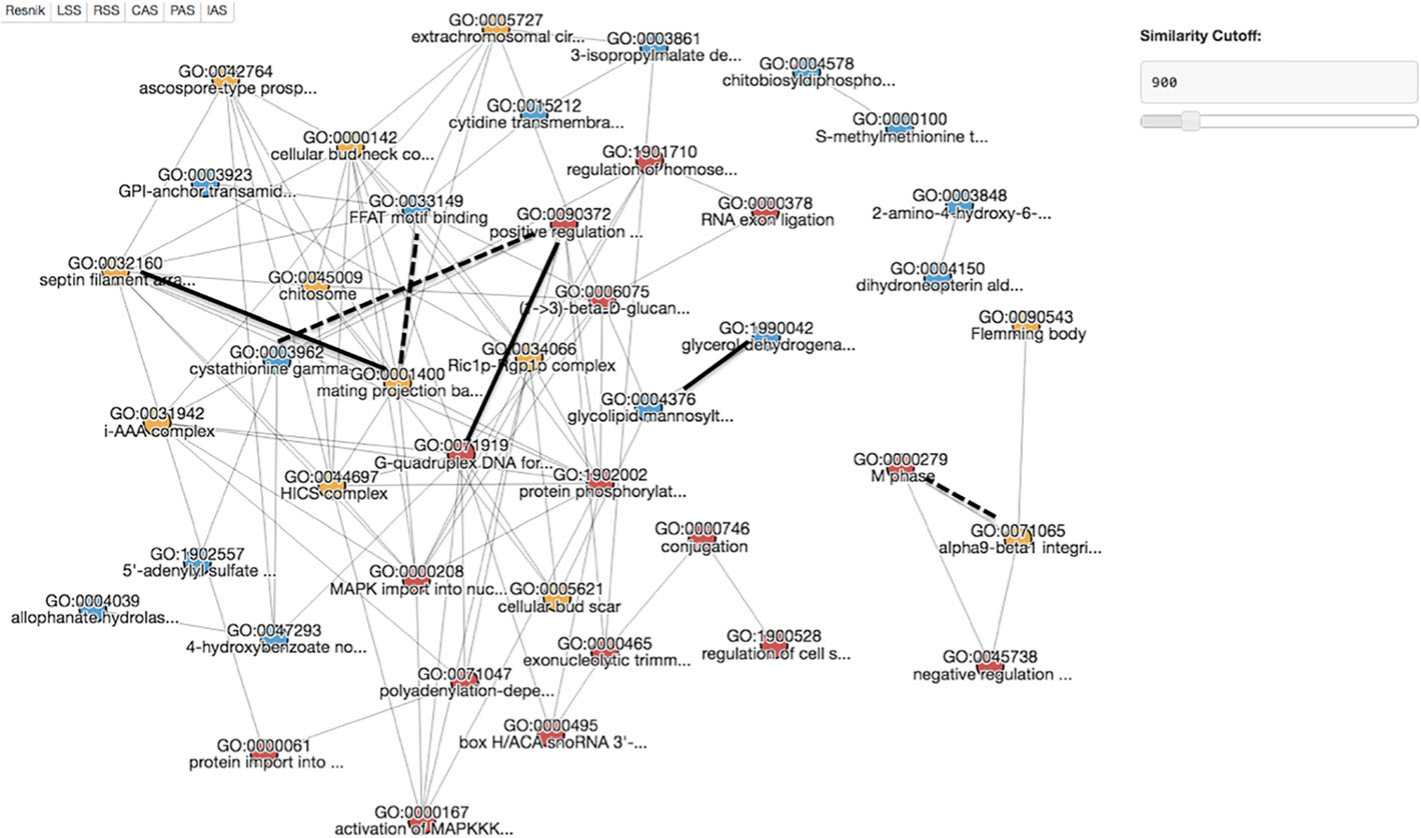
Network view of GO term association. A screenshot of the network view of GO term association in the resulted page. The score to consider can be switched by clicking the *bottom panel* on the *upper left corner*. GO terms from different GO categories are mapped in different colors (BP: *red*; MF: *blue*; CC: *yellow*). *Upper right corner* panel is used for adjusting a score cut-off threshold. Six GO pairs discussed as examples in Table 1 are highlighted in bold (*dashed lines* for cross-domain pairs)

**Fig. 4:**
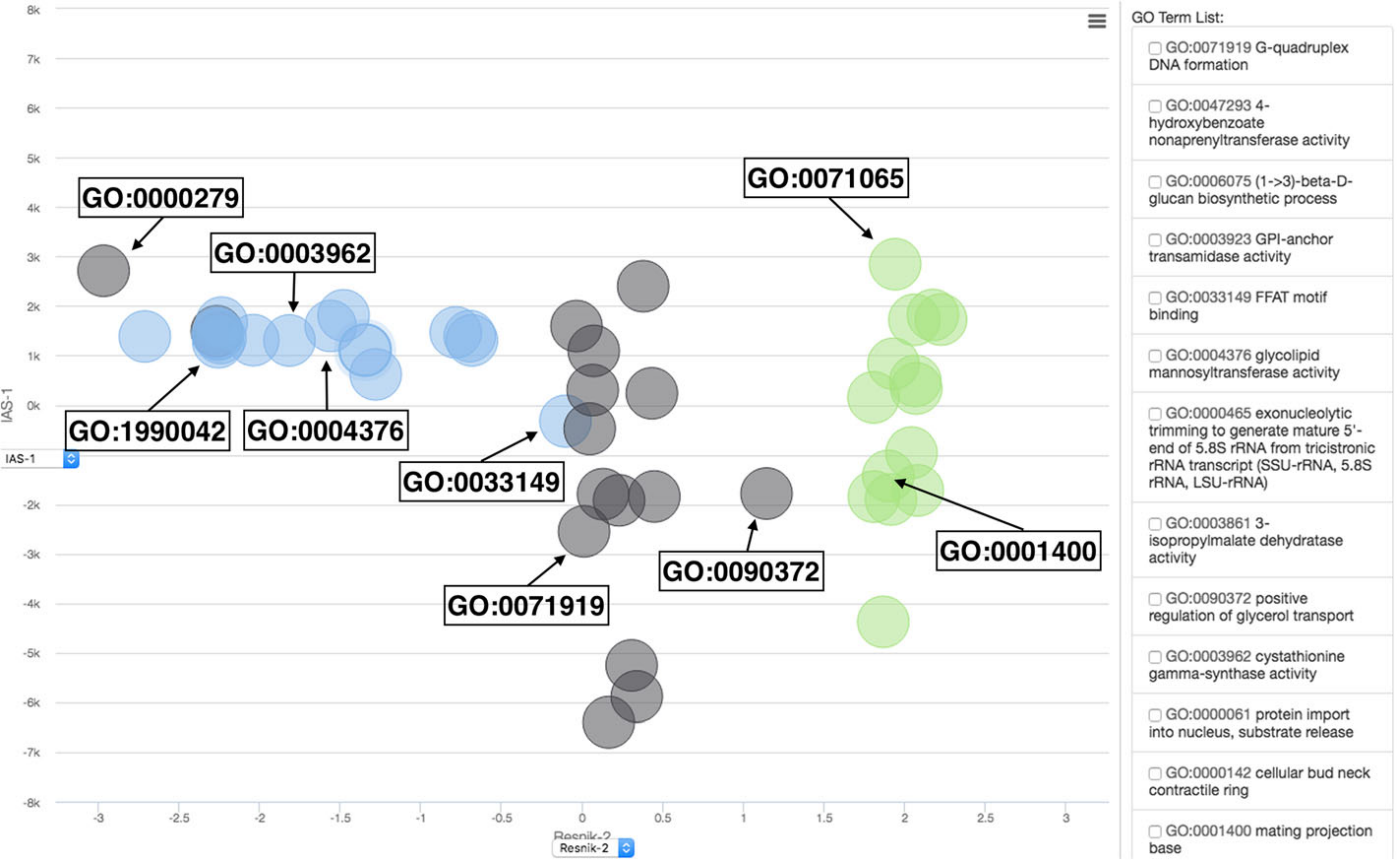
Bubble chart view of the GO term association. A screenshot of the bubble chart view in the resulted page. A bubble chart maps GO terms in the two dimensional space by considering their similarity in terms of two scores shown on the two axes. Score scheme to be used for each axis can be chosen by the option fields in the middle of the axis. Users can zoom in by holding down the mouse button and then dragging it to the desired area. The *right panel* shows GO terms that are currently visible on the chart. The example of shown here are visualization of the same set of 48 GO terms as used in Fig. 3. In this example plot, the X-axis is the Resnik semantic similarity score and the Y-axis chosen is IAS. For illustration, in this figure GO terms are colored according to their GO category, MF, *blue*; BP: *black*; and CC: *green*. GO terms listed in Table 1 are labelled

**Fig. 5:**
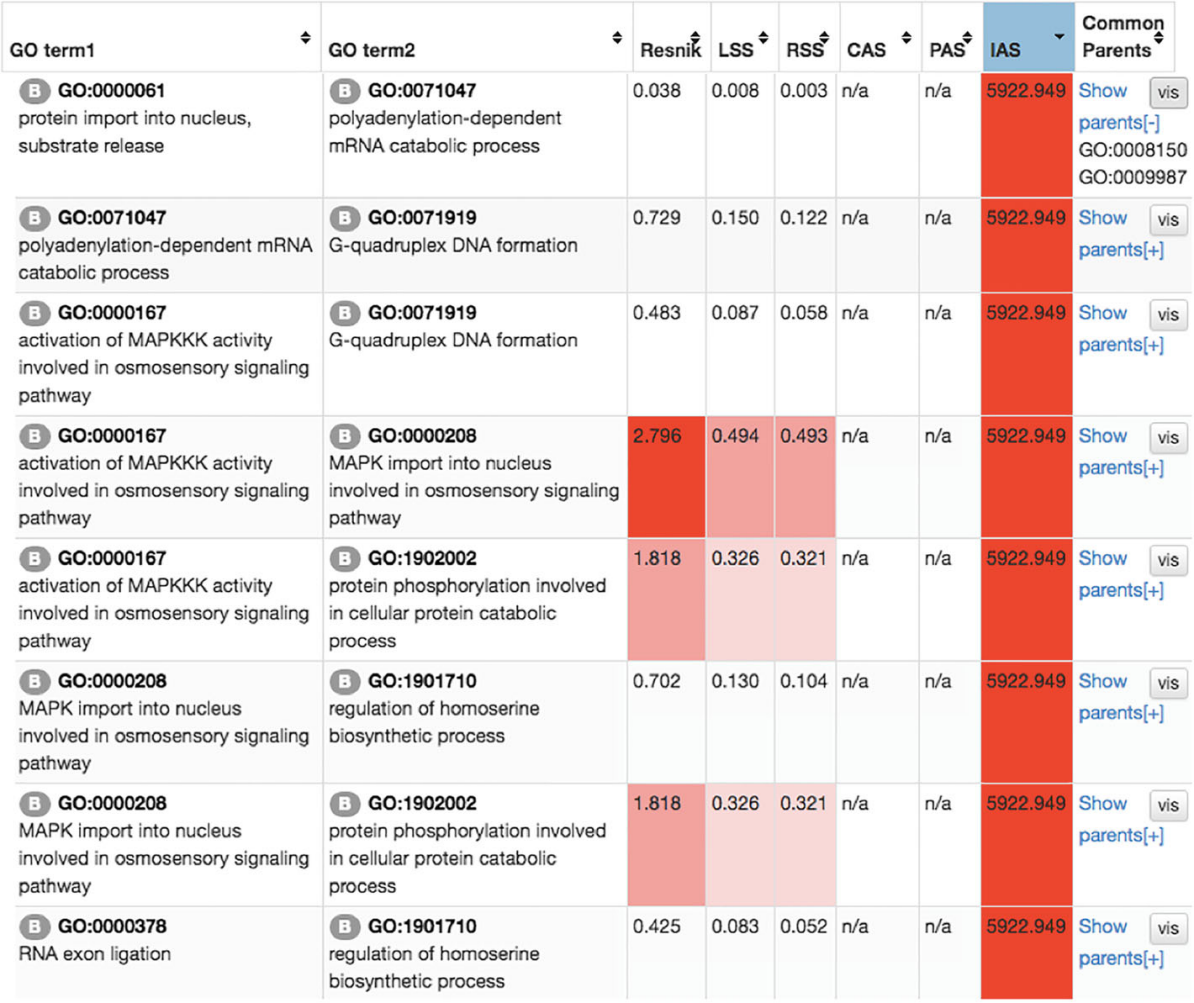
Table view of GO scores. The six different scores of all the GO term pairs are shown. CAS, PAS, and IAS of some GO term pairs are not available. This happens if the underlined statistics of the GO term pair was not sufficient at the time of the development of the CAS, PAS, and IAS. B in a circle at the left shoulder of a GO term indicates that the term belongs to the Biological Process (BP) category. Terms in Molecular Function and Cellular Component are labelled with M and C

The tool from the next tab “GO Set” computes pairwise GO association scores for a list of input GO terms and outputs them in three formats: a similarity graph (Fig. 3), a bubble chart (Fig. 4), and a score table (Fig. 5). From the result page, users can choose the three different output formats. For each type of GO scoring schemes described in Methods, four cutoff thresholds are provided, which were computed from the overall distribution of GO pair scores under each scheme (top 1%, 5%, 10%, and 20%) and shown in the color-scale (red to pink for high to low) in the table format (Fig. 5). The last column in the table contains common parents between GO pairs and a link to the interactive visualization with GO Visualizer (Fig. 2). The result table can be downloaded in a CSV format file.

Here for illustration, we used a set of 48 GO terms as input. From the GO pairs from the 48 GO terms, Table 1 lists six pairs where GO pairs has a high IAS but a low SS score. IAS indicates likelihood that protein pairs with high IAS GO terms have physical interaction, and thus different from functional similarity represented by SS scores. These six pairs are highlighted in bold in the network view of the GO terms in Fig. 3 (dashed lines for cross-domain pairs). The first three pairs in Table 1 have high IAS from the same GO category while the last three GO pairs in the table are pairs from different categories. The first example from the first group is GO:0071919 *G-quadruplex DNA formation* and GO:0090372 *positive regulation of glycerol transport*, both in BP. Only one common ancestral GO terms in the hierarchy (Fig. 2), GO:0008150 *biological process* is found for this GO term pair at the depth of 0. Since the lowest common ancestor of this pair is too shallow (i.e. general) in the GO hierarchy, the SS score for this pair is low (0.003). On the other hand, due to the large number of occurrence in interacting proteins in PPI, the IAS score of this GO pair is very high (2961.47).

**Table 1:** Examples of IAS that are different from the SS scores

GO ID 1	Description	Domain	GO ID 2	Description	Category	IAS	RSS	LSS	Resnik
GO:0071919	G-quadruplex DNA formation	BP	GO:0090372	Positive regulation of glycerol transport	BP	2961.47	0.003	-0.014	-0.076
GO:0004376	Glycolipid mannosyltransferase activity	MF	GO:1990042	Protein histidine kinase binding	MF	5922.95	0.042	0.074	0.364
GO:0001400	Mating projection base	CC	GO:0032160	Septin filament array	CC	4230.68	0.002	0.008	0.039
GO:0000279	M phase	BP	GO:0071065	Alpha9-beta1 integrin-vascular cell adhesion molecule-1 complex	CC	5922.95	N/A	N/A	N/A
GO:0033149	FFAT motif binding	MF	GO:0001400	Mating projection base	CC	1692.27	N/A	N/A	N/A
GO:0090372	Positive regulation of glycerol transport	BP	GO:0003962	Cystathionine gamma-synthase activity	MF	987.16	N/A	N/A	N/A

The last three examples illustrate cases where IAS can identify related GO terms across GO categories. The first example is a pair of BP GO term GO:0000279 *M phase* and CC GO term GO:0071065 *alpha9-beta integrin-vascular cell adhesion molecule-1 complex*. The SS score of these two terms is not calculated since these terms are from different categories, and SS is only defined for pairs from the same GO category. However, the pair has a very high IAS score (5922.95) because of the large amount of protein interactions in PPI network have these terms (Table 1).

Relationships of GO terms can be visualized in two ways, in a network or in a bubble chart, which provides intuitive understanding of similarity and relationship of GO terms. Fig. 3 shows a GO term association graph of IAS for the 48 GO terms. In this graph, GO terms are connected if they have an IAS above the threshold value (900 in this example) that can be controlled by the scale bar on the right upper corner of the panel (Fig. 3). GO terms in different categories are shown in different colors (BP: red; MF: blue; CC: yellow). The edges in bold and dashed lines are those pairs that are discussed in Table 1.

The bubble chart maps GO terms in terms of two scores users’ choice on the X- and Y-axis (Fig. 4) by a statistical dimension-reduction method named multidimensional scaling (MDS) [20, 21] implemented in R [22]. In case GO terms have the identical score, the centers of the circles/dots of the terms are shifted by a small amount to a random direction to avoid complete overlap. The bubble chart is interactive and the coordinate data is exportable. In Fig. 4, interestingly, along the Resnik score (X-axis), the GO terms are clearly separated by their GO category visualized in each different color, because the Resnik score for GO terms of different categories is 0, i.e. not defined, thus very far between them. In contrast, IAS is defined even for terms across GO categories. Thus, some GO pairs across different categories, for example, GO pairs of (GO: 0000279 from BP, GO: 007165 from CC) and (GO: 0033149 from MF, GO: 0001400 from CC) shown in the bottom half of Table 1, are close when mapped on the IAS (Y-axis) but far in Resnik (X-axis).

### GO Enrichment analysis

The NaviGO server supports the analysis of GO term enrichment. For an input list of annotated genes, enriched GO terms in the genes relative to the fraction in the entire genome will be identified. This is useful for finding dominant common functions for a set of genes, which are identified, for example, by gene expression data or protein-protein interaction network. Thus, the analysis can aid to identify the associated proteins involved in certain function within an organism. The server will automatically identify the organism based on the UniProt ID [23] of the first input protein; however, users can specify the organism in the Organism window manually. NaviGO will connect to the UniProt database by using their RESTful service and automatically retrieve the organism information and the background GO annotation information of the organism.

**Fig. 6:**
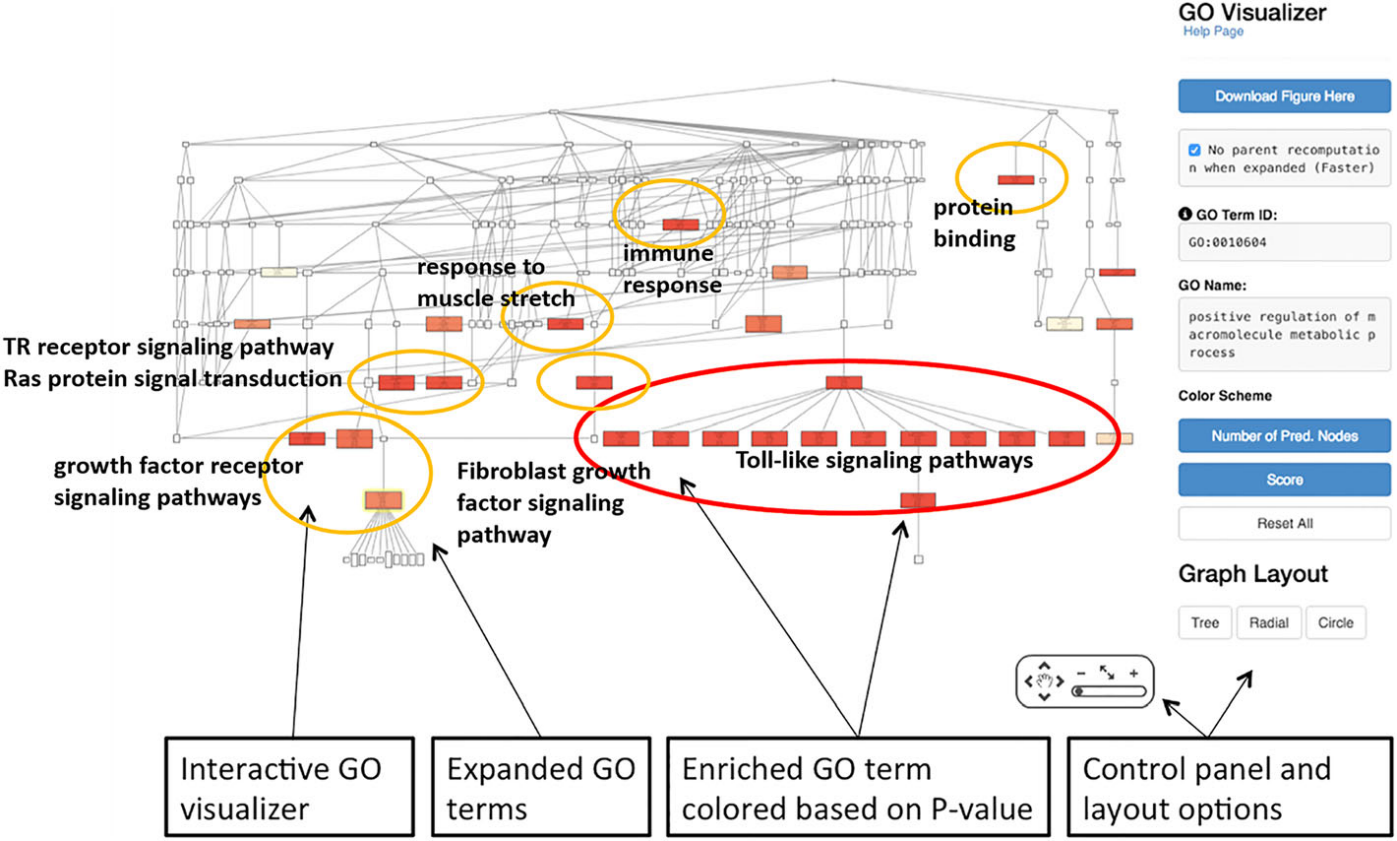
Visualization for enrichment analysis. The top 30 enriched GO terms from MAPK pathway proteins visualized in the GO hierarchy. Enriched GO terms are enlarged and colored by their *p*-value. The analysis is for 20 annotated proteins that are associated with MAPK signalling pathway. See text for the details

The result page lists GO terms sorted by calculated *p*-value. The *p*-value tells how rare (significant) it is to have enrichment of the GO term in the protein set considering the number of proteins in the set, the number of proteins with that GO term in the organism, and the number of proteins in the organism. GO terms of significant *p*-value (0.00005) (or top 30 GO terms, whichever smaller) will be visualized in the GO hierarchy (Fig. 6). The number of GO terms to visualize can be controlled manually by users. The enriched GO terms are color-mapped according to the *p*-value of enrichment on the GO DAG visualizer. In the example in Fig. 6, a GO enrichment analysis is shown for 20 annotated proteins that are involved in the MAPK signalling pathway, which were found in a SNP-targeted GWAS studies as set of proteins involved in the Rheumatoid Arthritis disease [24]. Enrichment analysis helps to identify the GO terms that are prominent among these disease associated proteins. GO terms, such as GO:0051403 *stress-activated MAPK cascade* with *p*-value of 4.04E-11 and GO:0000186 *activation of MAPKK activity* with *p*-value of 3.13E-9, are in the top enriched results. Due to the fact that the activation of the TLRs signalling pathway can trigger the activation of the MAPK pathways [24], GO terms like GO:0034166 *toll-like receptor 10 signaling pathway* also has a low *p*-value (5.06E-13) and ranked the top in the list (indicated with red circle). In Fig. 6, GO terms which has e-value of less than 1.0E-10 are circled in orange and their function descriptions are shown.

### Quantifying functional association of proteins

**Fig. 7:**
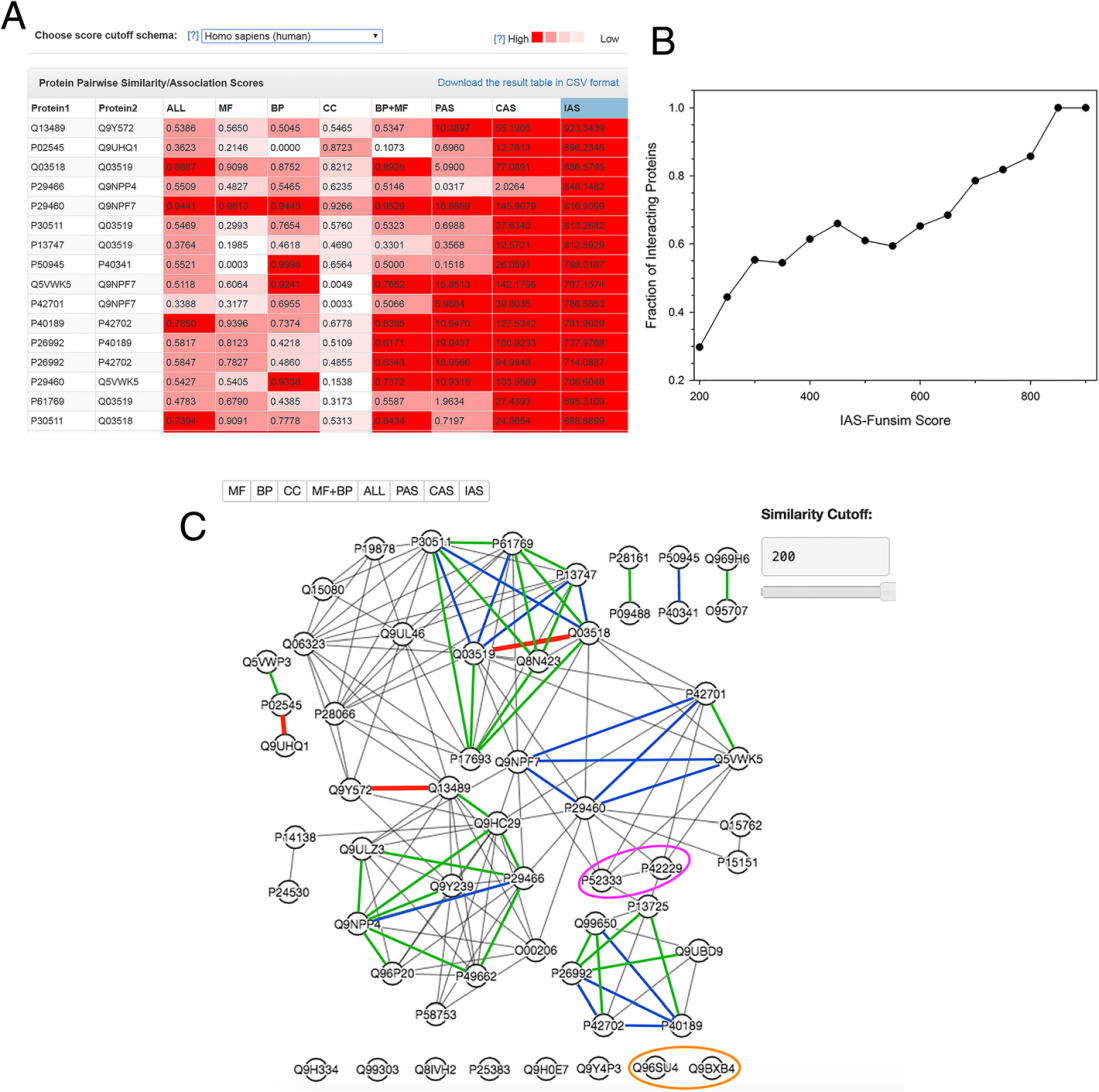
Example of the protein set analysis. Pairwise IAS scores among all protein pairs in the human genome were computed. **a**, A snap shot of the table of protein pairs in the result page sorted by the IAS score. The color level shows the significance of the scores. **b**, The fraction of the protein pairs that are actually physically interacting among those above IAS score cutoffs (x-axis). Pairs that have a score of 200 or higher are considered. Physically interacting protein pairs were checked with the BIOGRID database. For example, 100% of pairs that have a score of 850 or higher and 78.5% of pairs with a score of 700 or higher actually interact with each other. **c**, A network view of the 56 unique proteins from the top high-scoring 33 protein pairs. Protein pairs that have an IAS score of 200 or higher are connected by edges. Protein pairs that have a high IAS score of over 850, 650, and 450, are connected with thick color lines in red, blue, and green. There are three, 19, and 47 such pairs, respectively. Two protein pairs that are discussed in Table 2 are circled. The *magenta circle* shows an example of physically interacting pairs and the *circle* in *orange* shows a functionally similar protein pair that do not physically interact with each other, which is correctly identified with a low IAS. See text for more details

This functionality available from the “Protein Set” tab in the NaviGO website, takes a list of annotated proteins as input and computes functional relevance between each protein pair. This function help identifying functional groups in a set of proteins. The server can take a function annotation file in the CAFA format. The result will be provided in a table as well as an interactive clustering view. An example result shown in Fig. 7 are for the 33 protein pairs that have high IAS mong all the protein pairs from the human genome. Since IAS are defined for GO term pairs taken from physically interacting proteins, protein pairs with a high IAS are highly likely to be interacting with each other. In the result table (Fig. 7a), significance of similarity predictions is classified into five levels shown in a color-scale (red to pink for high to low). The scale indicates that the score is within top 1, 5, 10 and 20%, which are computed relative to the score distribution of all the protein pairs of the organism chosen by the user at the pull-down menu. The median option in the pull-down menu shows significance based on the average values of 5^th^ and 6^th^ genomes (i.e. median) when the 10 genomes in the list are sorted in the descending order of their corresponding cut-off values. The full result table is available to download in the CSV format.

In Fig. 7a, pairs are sorted by IAS. Notice that pairs with high IAS do not always have significant scores in terms of the other scores, e.g. ALL (functional similarity of proteins using Relevance Similarity scores (Eq. 6), indicating that IAS captures a unique feature of GO annotations of proteins, which are different from functional similarity. Figure 7b shows the fraction of physically interacting protein pairs among pairs with IAS above cutoffs. The graph shows that IAS has a substantial correlation to the fraction of interacting proteins, indicating IAS indeed detects physically interacting protein pairs. For example, all three protein pairs with a score above 850 actually interact with each other, and 68.4% (13 pairs) among 19 pairs that have an IAS over 650 have physical interactions between each other. Figure 7c provides the network view of the 33 protein pairs, where nodes are proteins and edges indicate pairs with IAS above a custom similarity cut-off value (200 is used). Pairs with significant scores, 850, 650, and 450, are highlighted in, red, green, and blue, respectively.

In Fig. 7c, two protein pairs are circled for discussion. These two pairs are listed in Table 2, a pair of P52333 and P42229 and another one, Q96SU4 and Q9BXB4. In the first pair, P52333 is a kinase that phosphorylates and the second one is STAT protein (P42229), which are involved in signal transduction and activation of transcription. These two proteins are not similar in function but physically interact with each other according to the BIOGRID database [25]. In contrast, the second pair, Q96SU4 and Q9BXB4 (orange circle in Fig. 7c), have high functional similarity (0.9033, right column), since they are both oxysterol-binding proteins (OSBP)-related proteins. However, as suggested with a low *funsim* IAS score (11.7716), they do not interact. These two pairs illustrate that *funsim* of IAS, which indicates possibe protein interactions, are different from conventional functional similarity. As shown here, by changing the underlying score for computing *funsim* protein pair score, we can see how proteins are similar or distinct in different aspects of GO term relationships.

**Table 2:** Comparison of Protein-pair Scores

Organism	Protein1	Function	Protein2	Function	Funsim-IAS score	Funsim-RSS score
Homo sapiens	P52333	Tyrosine-protein kinase JAK3	P42229	STAT 5A	395.6000	0.2879
Homo sapiens	Q96SU4	Oxysterol-binding protein-related protein 9	Q9BXB4	Oxysterol-binding protein-related protein 11	11.7716	0.9033

### Analysing function prediction results with NaviGO

NaviGO is linked from two function prediction servers, PFP [12, 13] and ESG [14], so that predicted GO terms for a gene by the servers can be easily further analysed. These two servers were ranked among top in gene function prediction contests held in recent years, the function prediction category in the Critical Assessment of techniques in protein Structure Prediction (CASP7) [26] and in the two rounds of the Critical Assessment of Function Annotation (CAFA) [27, 28]. PFP and ESG are available at http://kiharalab.org/pfp.php and http://kiharalab.org/esg.php [8]. Function prediction have become increasingly important because a substantial fraction of genes in a genome are unannotated [13].

**Fig. 8:**
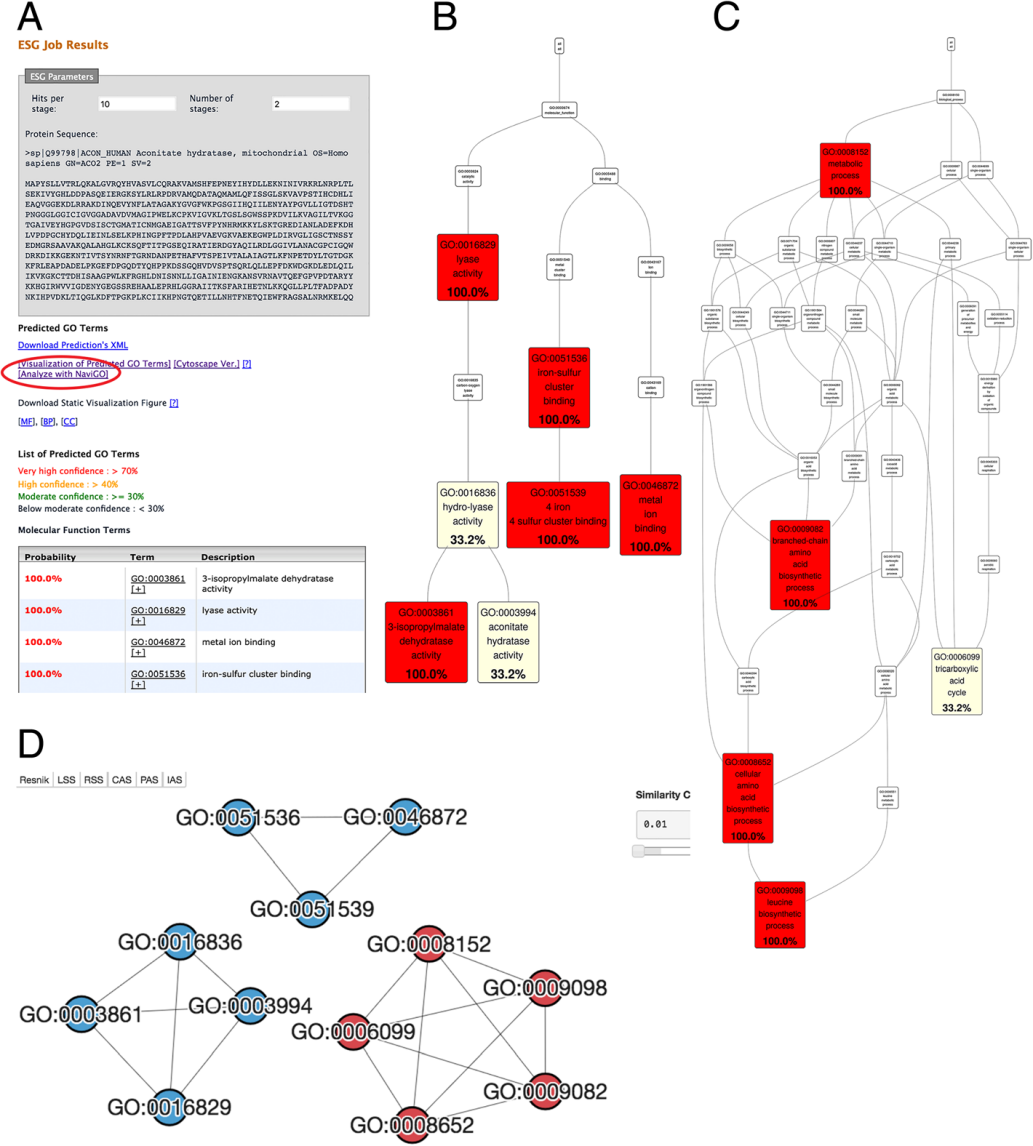
Analysing function prediction results with NaviGO. NaviGO is linked from the PFP and ESG function prediction webservers, which predict GO terms for input protein sequence. This example shows function prediction for human aconitate hydrolase (UniProt ID: Q99798). This protein is a moonlighting protein, which has two distinct function, aconitase and involvement of iron homeostatis. **a**, An output page of ESG. The output page has a link to NaviGO, which is indicated by a red circle in the figure. Clicking this link will send predicted GO terms of the query protein listed below in the table, which has the medium confidence or higher, to NaviGO’s GO Set input page, so that users can further analyse the predicted GO terms. **b**, Predicted GO terms in the MF category visualized in NaviGO. Color codes shows the confidence of prediction. **c**, Predicted GO terms in the BP category visualized in NaviGO. **d**, The network view of the predicted GO terms in NaviGO using the RSS, showing functionally similar GO terms in clusters. GO terms in MF and BP are colored in *blue* and *red*, respectively. We see two clusters for MF, indicating that this protein has two distinct functions

In the output page of PFP and ESG, where predicted GO terms are listed with confidence scores, the GO terms will be sent to NaviGO by clicking a link “Analyze with NaviGO” (Fig. 8a). Here we show a case that the analysis of predicted function by NaviGO revealed that a query protein has two distinct functions. The function prediction was performed by the ESG server for human aconitase (UniProt ID: Q99798). This protein is known as a moonlighting protein, which has two distinct independent functions [29, 30]. The primary function of this protein is an enzyme as aconitase while it is also known to be involved in iron homeostasis [29, 30]. Visualization of the predicted GO terms by NaviGO shows that the predicted GO terms in the MF category (Fig. 8b) are indeed separated in two branches, one on the left with the enzymatic activity (including lyase activity, aconitase hydrolase) and another branch on the right, iron-sulfer clustering binding, which is related to iron homeostasis (Fig. 8c), showing that the prediction correctly captured two distinct functions of this protein. The network view of the predicted GO terms also clarified that the protein has two distinct MF functions (shown in blue nodes), indicated by the two clusters.

## Conclusions

A web-based tool for analysing GO terms and gene annotation was developed. Results are visualized by a user-friendly interactive panel, which provides intuitive understanding of gene function. A strength of NaviGO is that similarity or association of GO terms can be quantified in six different scores and it is equipped with real-time rendering of GO terms in the GO hierarchy. The unique feature of NaviGO should provide great convenience in functional analysis with GO for both bioinformatics researchers and biologists.

## Methods

NaviGO is as a web-based tool at http://kiharalab.org/web/navigo. The source codes are made available at Github, https://github.com/kiharalab/NaviGO and https://github.com/kiharalab/GOVisualizer.

### GO similarity/association scores

In NaviGO, six scores can be used to quantify similarity or association relationship of GO terms. Three scores are for quantifying semantic similarity of GO terms: Resnik’s, Lin’s, and the relevant semantic similarity score. The other three scores, CAS, PAS, and IAS are for quantifying GO associations. Detailed explanation of the scores is provided in separate sections below.

To quantify the functional similarity of two genes, the *funsim* score [4, 5] is used. Funsim of two sets of terms, i.e. GO annotations of two genes, is calculated from an all-by-all similarity matrix, where each entry of the matrix is a similarity score of users’ choice between a GO pair.

### CAS and PAS

We previously developed two function association scores, Co-occurrence Association Score (CAS) and PubMed Association Score (PAS) [3]. CAS quantifies frequency of co-occurring GO terms within the gene annotations in the GOA database while PAS takes consideration of co-occurrence of GO terms in PubMed abstracts. A characteristic differentiating the two methods from other methods is that the two scores can be defined cross-domain associations between GO terms, i.e. terms from Molecular Function (MF) and Biological Process (BP), those from MF and Cellular Component (CC), and those from BP and CC.

1 $$ C A S\left( i, j\right)=\frac{\frac{c\left( i, j\right)}{{\displaystyle {\sum}_{ij} c\left( i, j\right)}}}{\left(\frac{c(i)}{{\displaystyle {\sum}_k c(k)}}\right)\left(\frac{c(j)}{{\displaystyle {\sum}_k c(k)}}\right)} $$

where *C(i,j)* is the number of sequences in the database that contain both the GO terms *i* and *j*. Similarly, *C(i)* is the total number of sequences annotated with the GO term *i*, and so is the *C(j)*. The numerator of Eq. 1, $\frac{c\left( i, j\right)}{{\displaystyle {\sum}_{ij} c\left( i, j\right)}}$ is essentially the fraction of sequences that are annotated with two particular GO terms, *i* and *j*, among all the sequences in the database. The denominator multiplies the fraction (probability) of sequences in the database that are annotated with GO term *i* and the fraction of sequences in the database that are annotated with GO term *j*. Thus, it is the expected fraction of sequences in the database with the two GO annotations, *i* and *j*, if i and j are randomly assigned to sequences. Using the numerator and the denominator, altogether CAS quantifies how often two GO terms *i* and *j* co-annotate sequences relative to the random chance. CAS = 1 means that the observation of co-annotation of *i* and *j* is the same as expected by the random chance, and a larger value indicates that *i* and *j* are correlated in gene annotation.

Similarly, PAS is defined as:

2 $$ P A S\left( i, j\right)=\frac{\frac{Pub\left( i, j\right)}{{\displaystyle {\sum}_{i, j}}\; Pub\left( i, j\right)}}{\left(\frac{Pub(i)}{{\displaystyle {\sum}_k}\; Pub(k)}\right)\left(\frac{Pub(j)}{{\displaystyle {\sum}_k}\; Pub(k)}\right)}=\frac{Pub\left( i, j\right)}{Pub(i) Pub(j)}\cdot \frac{{\left({\displaystyle {\sum}_k} Pub(k)\right)}^2}{{\displaystyle \sum {{{}_k}_{,}}_l}\; Pub\left( k, l\right)} $$

Here, *Pub(i,j)* is the number of PubMed abstracts which contain both the GO terms *i* and *j*. Similarly, *Pub(i)* is the number of abstracts that contain GO term *i* and the same is applicable for *Pub(j)*. The numerator of Eq. 2, $\frac{Pub\left( i, j\right)}{{\displaystyle {\sum}_{ij\;} Pub\left( i, j\right)}}$, is the fraction of abstracts in PubMed that mention two particular GO terms, *i* and *j*, among all the abstracts in the PubMed database. The denominator multiplies the fraction (probability) of abstracts in PubMed that mention GO term *i* and the fraction of abstracts that mention GO term *j*. Thus, it is the expected fraction of abstracts in the database with the two GO annotations, *i* and *j*, if i and j randomly show up in abstracts. Altogether, PAS quantifies how often two GO terms *i* and *j* are co-mentioned in PubMed abstracts relative to the random chance. PAS = 1 means that GO term *i* and *j* are not related, and a larger value indicates that *i* and *j* are related and frequently co-mentioned in biological contexts. Importantly, it is possible that GO terms that do not have a high functional similar scores (Resnik, Lin’s, and Relevance Similarity scores) have a high CAS or PAS. High PAS and CAS implies that proteins with the GO term annotation are functionally related and play roles in the same biological context, e.g. pathways.

### IAS

**Fig. 9:**
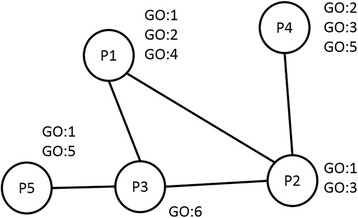
Computation of IAS. An example of a PPI network from which IAS of GO term pairs can be computed. Five proteins, P1 to P5, are in this network, and edges indicate that connected protein pairs interact with each other. GO annotation of each protein is listed next to the protein

The Interaction Association Score (IAS) [7] captures the propensity of GO term pairs to occur in interacting proteins by counting the number of GO term pair that occur in interacting proteins normalized by random chance. Thus, high IAS between a protein pair indicates a high possibility that the protein pairs interact with each other. The GO_IAS for each GO term pair was computed as follows:

3 $$ GO\_ I A S\left( GOx, GOy\right)=\frac{\frac{N\left( GOx, GOy\right)}{\# T. Edges}}{\left(\frac{N(GOx)}{\# T. Nodes}\right)\left(\frac{N(GOy)}{\# T. Nodes}\right)} $$

where *N(GOx-GOy)* is the number of times GO term pair *GOx* and *GOy* interact in PPI networks, *#T.Edges* is the total number of interactions (edges) in PPI networks, *N(GOx)* and *N(GOy)* are the number of times *GOx* and *GOy* independently occur in proteins the networks, and *#T.Nodes* is the total number of proteins in the PPI networks. Figure 9 shows an example of a small PPI network. This network has 5 edges between 5 proteins; 3 proteins are annotated with GO:1, and 2 proteins with GO:2. There are 2 edges that connects between GO:1 and GO:2 (P1 to P2 and P2 to P4). From this network, GO_IAS for GO:1 and GO:2 is computed as (2/5)/((3/5)(2/5)) = 1.67. Similar to PAS and CAS, IAS quantifies how often two GO terms *i* and *j* are observed in physically interacting proteins in a protein-protein interaction network relative to the expected number of observations by the random chance. If two proteins are annotated with GO terms that have high IAS, it suggests that the proteins may physically interact with each other.

Significant difference between CAS, PAS, and IAS from conventional GO functional similarity scores described in the next section is that the former three scores quantifies functional relevance of GO term pairs in biological contexts, co-annotation to genes (CAS), co-mention in PubMed abstracts (PAS), and interacting protein pairs (IAS). Due to the design, these scores are capable of identifying proteins in the same pathways (CAS, PAS) [3] and physical interacting proteins (IAS) [7]. Correlation of CAS/PAS/IAS to regular functional similarity scores (below) is not very high [3, 7], because proteins in the same pathway and physically interacting proteins are not necessarily have similar function.

### Resnik, Lin’s, and relevance similarity scores

For quantifying GO term similarity, NaviGO provides three score options. The Resnik’s [5] similarity score measures the semantic similarity of a GO term pair according to the lowest common ancestor (LCA) of the GO term pair, while the Lin’s similarity is based on the information content of LCA and the GO term pair queried [3].

4 $$ s i{m}_{Resnik}\left({c}_1,{c}_2\right)={ \max}_{c\in S\left({c}_1,{c}_2\right)}\left(- \log p(c)\right) $$

5 $$ s i{m}_{Lin}\left({c}_1,{c}_2\right) = { \max}_{c\in S\left({c}_1,{c}_2\right)}\left(\frac{2\cdot log\  p(c)}{log\  p\left({c}_1\right)+ log\  p\left({c}_2\right)}\right) $$

Here *p(c)* is the probability of a GO term c, which is defined as the fraction of the occurrence of *c* in the GO Database. *s(c1,c2)* is the set of common ancestors of the GO terms c1 and c2. The root of the ontology has a probability of 1.0.

The relevance semantic similarity score (sim_Rel_) [4] for computing functional similarity of a pair of GO terms, c1 and c2:

6 $$ s i{m}_{Rel}\left({c}_1,{c}_2\right) = { \max}_{c\in S\left({c}_1,{c}_2\right)}\left(\frac{2\cdot log\  p(c)}{log\  p\left({c}_1\right)+ log\  p\left({c}_2\right)}\cdot \left(1- p(c)\right)\right) $$

The first term considers the relative depth of the common ancestor c to the average depth of the two terms c1 and c2 while the second term takes into account how rare it is to identify the common ancestor c by chance.

### Functional similarity score of gene pairs

To quantify the functional similarity of two annotated genes, we used the *funsim* score [4, 5]. The *funsim* score of two sets of terms, GO^A^ and GO^B^ for gene A and B, of a respective size of N and M, is calculated from an all-by-all similarity matrix *s*_*ij*_.

7 $$ S i j= s i m{\left( G{O_i}^A, G{O_j}^B\right)}_{\forall i\in \left\{1.. N\right\},\forall j\in \left\{1.. M\right\}} $$

For *sim(GO*_*i*_^*A*^ *, GO*_*i*_^*B*^ *)*, the relevance similarity score is usually used but other scores can be used, too. Since the relevance similarity score is defined only for GO pairs of the same category, a matrix is computed separately for the three categories, BP, CC, and MF:

8 $$ G{O}_{s core}= \max \left(\frac{1}{N}{\displaystyle \sum_{i=1}^N\underset{1\le i\le M}{ \max }{s}_{i j}}\right.,\left.\frac{1}{M}{\displaystyle \sum_{i=1}^M\underset{1\le i\le N}{ \max }{s}_{i j}}\right) $$

GOscore will be any of the three category scores (MFscore, BPscore, CCscore). Finally, the *funsim* score is computed as

9 $$ funsim=\frac{1}{3}\left[{\left(\frac{MFscore}{ \max (MFscore)}\right)}^2+{\left(\frac{BPscore}{ \max (BPscore)}\right)}^2+{\left(\frac{CCscore}{ \max (CCscore)}\right)}^2\right] $$

where *max(GOscore)* = 1 (maximum possible GOscore) and the range of the *funSim* score is [0, 1].

### Gene ontology enrichment analysis

The probability of a GO term X being annotated to a protein in the cluster is computed by:

10 $$ f\left( k; N,\  m, n\right)=\frac{\left(\begin{array}{c}\hfill m\hfill \\ {}\hfill k\hfill \end{array}\right)\left(\begin{array}{c}\hfill N- m\hfill \\ {}\hfill n-\mathrm{k}\hfill \end{array}\right)}{\left(\begin{array}{c}\hfill N\hfill \\ {}\hfill n\hfill \end{array}\right)} $$

where *k* is the number of proteins in the cluster annotated with X, *N* is the number of annotated proteins in the organism, *m* is the number of proteins in the organism annotated with X, and *n* is the number of annotated proteins in the cluster. To calculate a *p*-value for overrepresentation of a term, we use the following equation:

11 $$ {P}_{hg}(X)={\displaystyle {\sum}_{i= k}^n f\left( i; N, m,\mathrm{k}\right)} $$

## Availability and requirements

**Project name**: NaviGO

**Project home pages**: http://kiharalab.org/web/navigo, https://github.com/kiharalab/NaviGO, and https://github.com/kiharalab/GOVisualizer

**Operating system(s)**: Web application, platform independent

**Porgram language**: Perl, Python 2.7, Python 3.4, and Ruby

**Other requirements**: Installation of a MySQL server

**Licence**: Source codes are released under the terms of the GNU Lesser General Public License Ver.2.1

## Abbreviations

BP: Biological process
CAS: Co-occurrence association score
CC: Cellular component
DAG: Directed acyclic graph
GO: Gene ontology
IAS: Interaction association score
MDS: Multi-dimensional scaling
MF: Molecular function
PAS: PubMed association score
PPI: Protein-protein interaction
RSS: Relevance semantic similarity
SS: Semantic similarity
